# Electrospun Janus-like pellicle displays coinstantaneous tri-function of aeolotropic conduction, magnetism and luminescence†

**DOI:** 10.1039/c9ra06444c

**Published:** 2019-09-30

**Authors:** Yunrui Xie, Qianli Ma, Haina Qi, Yan Song, Jiao Tian, Makiyyu Abdullahi Musa, Wensheng Yu, Xiangting Dong, Dan Li, Guixia Liu

**Affiliations:** Key Laboratory of Applied Chemistry and Nanotechnology at Universities of Jilin Province, Changchun University of Science and Technology Changchun 130022 China dongxiangting888@163.com +86 431 85383815 +86 431 85582575

## Abstract

A new Janus-like pellicle with top-bottom structure, functionalized by conductive aeolotropism, magnetism and luminescence (defined as a CML Janus-like pellicle), is conceived and constructed *via* electrospinning by combining microcosmic with macroscopic partitions. [PANI/PMMA]//[Eu(BA)_3_phen/PMMA] and [Fe_3_O_4_/PMMA]//[Tb(BA)_3_phen/PMMA] Janus-like microribbons are selected as building units to construct a conductive aeolotropism-luminescence layer (CL layer) and magnetism-luminescence layer (ML layer), and the two layers are combined to form a CML Janus-like pellicle. Macroscopic partition is achieved by designing the Janus-like structure of the pellicle, while Janus-like microribbons are used for the microcosmic partition by separating rare earth luminescent compounds from dark-colored magnetic Fe_3_O_4_ NPs and conductive PANI. The CML Janus-like pellicle has stronger luminescence compared to the contrast samples. The magnetism of the CML Janus-like pellicle can be adjusted by changing the doping amount of Fe_3_O_4_ NPs. The CML Janus-like pellicle can achieve a strong and variable conductive aeolotropism *via* changing the doping amount of PANI and the highest conductive aeolotropism ratio can reach *ca.* 10^8^ times when the PANI content is 70%. Microcosmic and macroscopic partitions are simultaneously integrated into the CML Janus-like pellicle, which results in almost no detrimental mutual influences between the two layers, and the overall performances of the CML Janus-like pellicle are greatly improved.

## Abstract 2

Janus-like pellicle with conductive aeolotropism, magnetism and luminescence is designed and constructed *via* electrospinning by combining microcosmic with macroscopic partitions.

## Graphical abstract

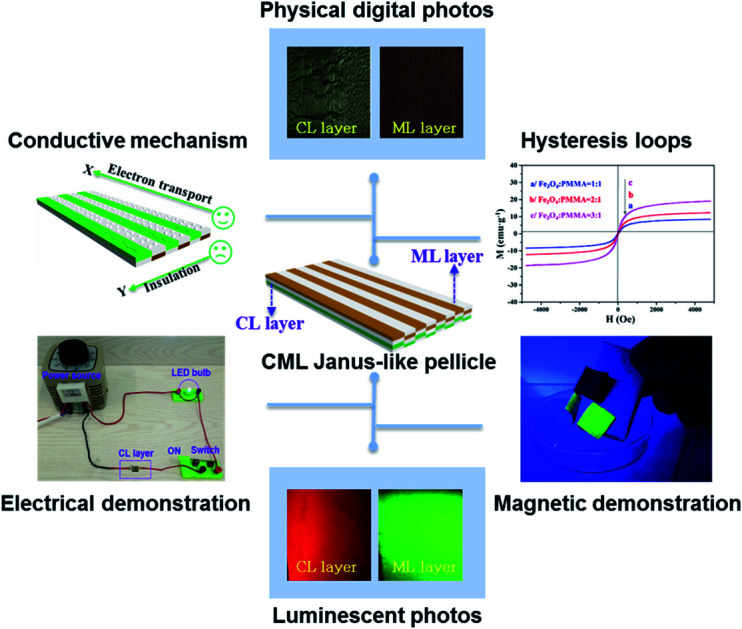

## Introduction

As novel materials, multifunctional materials that synchronously have two or more exceptional performances have drawn great attention due to special structures and significant applications in many areas, such as imaging,^1–3^ oil-water separation,^4,5^ optoelectronic devices^6,7^ and environment.^8,9^ There are many methods to obtain multifunctional materials, such as chemical grafting,^10^ modification^11,12^ and compound methods.^13,14^ Compound is one of the universal methods to obtain multifunctional materials with comprehensive performances. Introducing different substances into a matrix^15,16^ is a typical compound method. However, an obvious shortcoming is mutual interferences between different substances, so it is necessary to design effective and novel micro-structures and macro-structures to separate different substances, and ensure different functions of the materials.^17,18^ For multi-functional nanomaterials, different building units such as Janus-like nanoribbons and Janus-like nanofibers not only lead to the microscopic separation of different substances to reduce negative interferences, but also ensure the multi-functionality of materials. Macroscopically, materials are often designed as layered structures^19–21^ in order to further reduce interference, such as two-layer structure,^22–25^ sandwich structure^26,27^ or multi-layer composite structure.^28–30^ Different layers have independent areas and are highly and effectively integrated into a material. All layers in the material can choose to work cooperatively or independently in accordance with practical application requirements. The structural design of multifunctional nanomaterials has attracted wide attention, and many research teams are committed to design reasonable micro-structure and macro-structure to acquire more excellent performances.^31–33^

Microscopic partition^34,35^ can greatly reduce negative influences among different substances, so it is significant to design reasonable micro-structure to achieve microscopic partition of multifunctional nanomaterials, such as bistrand aligned composite nanofiber,^36^ peculiarly structured Janus nanofiber,^37,38^ Janus nanofiber^39^ and Janus-like nanoribbons.^40–42^ Janus-like nanoribbons is a typical micro-structure to microscopically separate different substances. The luminescence intensity of nanomaterials is evidently reduced owing to the absorption of light when luminescent substances and dark substances are directly mixed together.^43,44^ Thus, Janus-like nanoribbons are often used to separate luminescent substances from dark substances to optimize overall performances of materials. Reasonable and outstanding macro-structure is also devised in order to further gain macroscopic partition and avoid negative interferences. As a novel macro-structure, Janus-like pellicle is one of effective and practical methods to obtain macroscopic partition. The structure of Janus-like pellicle can be classified as top-bottom and left-right structure. Janus-like pellicle, with top-bottom structure, has attracted wide attention due to unique and asymmetric structures and properties.^45,46^ The work patterns of Janus-like pellicle with top-bottom structure are mainly divided into two modes. First one, the top layer and bottom layer must collaboratively work and the pellicle will lose multifunctionality without either layer, such as directional transport Janus pellicle. This work pattern is described as synergistic mode. Second one, the top and bottom layers can work collaboratively or independently according to the actual requirements. This work pattern is described as individual mode.^47^ The appropriate functional layers can be selected according to practical applications, and there are no interferences among the functional layers. It can simultaneously implement functional partitioning and functional integration. Flexible applications of Janus-like pellicle can be achieved by choosing the appropriate work patterns according to specific requirements. Based on the above discussion and previous work of our group,^48,49^ we combine microcosmic partition with macroscopic partition, and select Janus-like microribbon as micro-building unit and Janus-like structure as macro-structure to realize effective separation of different substances, and obtain multi-functional materials with excellent properties.

In this work, a new Janus-like pellicle, functionalized by conductive aeolotropism, magnetism and luminescence (defined as CML Janus-like pellicle) with top-bottom structure, is constructed by combining microcosmic with macroscopic partitions. The layered structure of CML Janus-like pellicle realizes macroscopic partition, and the microcosmic partition is realized through Janus-like microribbon, thus realizing the high integration of microcosmic and macroscopic partitions in a material to obtain the integrity and versatility of the material. [PANI/PMMA]//[Eu(BA)_3_phen/PMMA] Janus-like microribbons and [Fe_3_O_4_/PMMA]//[Tb(BA)_3_phen/PMMA] Janus-like microribbons are separately used as building units to construct conductive aeolotropism-luminescence layer (named as CL layer) and magnetism-luminescence layer (marked as ML layer). The preparation of different layers and the assembly are achieved *via* electrospinning. The effective separations among luminescent, magnetic and conductive substances are successfully achieved by the microscopic Janus-like microribbons. The macroscopic Janus-like structure of pellicle further efficaciously avoids detrimental influences among different performances. The prepared CML Janus-like pellicle has high and adjustable conductive aeolotropism, adjustable magnetism and bicolored luminescence. Such multifunctional materials will play an important role in nanodevices, flexible electronic devices and so on.

## Experimental sections

### Chemicals

The related chemical reagents were summarized in the ESI.†

### Preparation of Eu(BA)_3_phen complexes, Tb(BA)_3_phen complexes, Fe_3_O_4_ nanoparticles (NPs) and Poly(methyl methacrylate) (PMMA)s

Eu(BA)_3_phen complexes, Tb(BA)_3_phen complexes, Fe_3_O_4_ nanoparticles (NPs) and Poly(methyl methacrylate) (PMMA) were synthesized in line with the ref. 18 and 43.

### Synthesis of spinning dopes

We explored the best ratios when different percentages of rare earth complex were doped into the same mass of PMMA to obtain the maximum luminescence intensity in previous related studies. In this study, the mass percentage of rare earth complex (Eu(BA)_3_phen and Tb(BA)_3_phen) to PMMA was fixed as 15%, as it was reported to give maximum luminescence intensity.^37,50^ Eu(BA)_3_phen (0.15 g) and PMMA (1.00 g) were put into a mixed solvent of DMF (1.00 g) and CHCl_3_ (10.00 g) under magnetic stirring for 24 h at room temperature (marked as spinning dope 1). Similarly, Tb(BA)_3_phen (0.15 g) and PMMA (1.00 g) were added into the mixed solvent of DMF (1.00 g) and CHCl_3_ (10.00 g) under magnetic stirring for 24 h at room temperature (called as spinning dope 2). In this work, we used PANI doped with camphor sulfonic acid (CAS) to provide electrical conductivity. PANI doped with CAS was synthesized while preparing conductive spinning dopes. Conductive spinning dopes were obtained through following process: firstly, a certain amount of ANI, CSA and PMMA (1.00 g) were dissolved into the mixture of DMF (1.00 g) and CHCl_3_ (10.00 g) under magnetic stirring for 48 h at room temperature (marked as solution A). Subsequently, a certain amount of APS was dissolved into DMF (2.00 g) and stirred for 1 h at room temperature (labeled as solution B). The two solutions were simultaneously frozen for 20 min at 0 °C in a refrigerator and the solution B was added into solution A by inches under magnetic stirring for 3 h in an ice-water mixture, and spinning dope 3 was formed. Then spinning dope 3 was refrigerated for 24 h at 0 °C. The color of spinning dope 3 changed from light brown to dark green, illustrating PANI doped with CAS was successfully prepared in this step. In order to achieve variable conductive aeolotropism, the mass percentage of PANI to PMMA was changed from 15%, 30%, 50% to 70%. The specific amounts of used materials of spinning dope 3 were summarized in Table 1. Magnetic spinning dopes were obtained through following process: a certain amount of Fe_3_O_4_ NPs was dispersed into the mixture of DMF (1.00 g) and CHCl_3_ (10.00 g) and ultrasonically dispersed for 20 min. Then 1.00 g of PMMA was added into the mixture under mechanical stirring for 24 h at room temperature, where spinning dope 4 was formed. The mass ratio of Fe_3_O_4_ NPs to PMMA was varied from 1 : 1, 2 : 1 to 3 : 1 to gain adjustable magnetism. The specific amounts of used materials of spinning dope 4 were summarized in Table 2.

**Table 1:** Compositions of the spinning dope 3

Spinning dope 3	PANI : PMMA (wt%)	ANI (g)	CSA (g)	APS (g)	PMMA (g)	DMF (g)	CHCl_3_ (g)
3-1	15	0.15	0.30	1.00	1.00	1.00	10.00
3-2	30	0.30	0.60	1.20	1.00	1.00	10.00
3-3	50	0.50	1.00	1.40	1.00	1.00	10.00
3-4	70	0.70	1.40	1.60	1.00	1.00	10.00

**Table 2:** Compositions of the spinning dope 4

Spinning dope 4	Fe_3_O_4_ : PMMA (mass ratio)	Fe_3_O_4_ (g)	PMMA (g)	DMF (g)	CHCl_3_ (g)
4-1	1 : 1	1.00	1.00	1.00	10.00
4-2	2 : 1	2.00	1.00	1.00	10.00
4-3	3 : 1	3.00	1.00	1.00	10.00

A mixed spinning solution (marked as spinning dope 5) of Eu(BA)_3_phen and PANI was prepared and the actual preparation process is as follows: a certain amount of ANI, CSA, Eu(BA)_3_phen and PMMA were added into the mixed solvent of DMF and CHCl_3_ under magnetic stirring (48 h) at room temperature. The mass percentage of PANI to PMMA was fixed as 15% for spinning dope 5. The amounts of substances and the other experimental procedures were same as those for preparing spinning dope 3. A mixed spinning solution (named as spinning dope 6) of Tb(BA)_3_phen and Fe_3_O_4_ NPs was prepared and the specific process was as follows: a certain amount of Fe_3_O_4_ NPs was ultrasonically dispersed into the mixed solvent of DMF and CHCl_3_ for 20 min. After that, Tb(BA)_3_phen and PMMA were put into the mixture under mechanically stirring for 24 h at room temperature. The mass ratio of Fe_3_O_4_ NPs to PMMA was designated as 1 : 1 for spinning dope 6. The amounts of related materials were same as spinning dope 4.

### Preparation of CML Janus-like pellicle

**Fig. 1:**
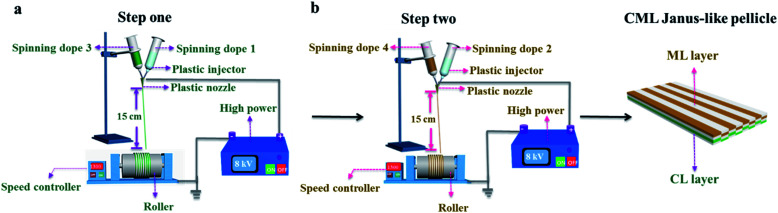
Schematic diagram of the fabrication of [PANI/PMMA]//[Eu(BA)_3_phen/PMMA] Janus-like microribbons array (named as CL layer) (a) and [Fe_3_O_4_/PMMA]//[Tb(BA)_3_phen/PMMA] Janus-like microribbons array (defined as ML layer) (b) of CML Janus-like pellicle.

[PANI/PMMA]//[Eu(BA)_3_phen/PMMA] Janus-like microribbons and [Fe_3_O_4_/PMMA]//[Tb(BA)_3_phen/PMMA] Janus-like microribbons are respectively selected as building units to construct CML Janus-like pellicle with top-bottom structure. Fig. 1 shows the schematic diagram of the preparation process of CML Janus-like pellicle. In the first step, 3 mL of the spinning dope 1 and 3 mL of the spinning dope 3 separately were dumped into two 5 mL plastic injectors with a parallel spinneret to obtain [PANI/PMMA]//[Eu(BA)_3_phen/PMMA] Janus-like microribbons array. The bottom layer of CML Janus-like pellicle was obtained after 3 h-electrospinning and named as CL layer. The roller was served as the collector and the diameter of roller was 10 cm. The CL layer did not need to be removed from the collector and the top layer is prepared directly on it. In the second step, 3 mL of the spinning dope 2 and 3 mL of the spinning dope 4 were separately dumped into two 5 mL plastic injectors with the parallel spinneret to obtain [Fe_3_O_4_/PMMA]//[Tb(BA)_3_phen/PMMA] Janus-like microribbons array. The top layer of the CML Janus pellicle was obtained after 3 h-electrospinning and defined as ML layer. The distance between the tip of plastic nozzle and the roller was set to 15 cm during the two steps. The applied voltage was 8 kV and the rotating speed of the roller was fixed as 1300 rpm throughout the preparation process. The thicknesses of CL layer and ML layer in CML Janus-like pellicle respectively were 185 μm and 191 μm. The actual temperature and relative air humidity respectively were 24 °C and 25% throughout the process.

### Fabrication of contrast samples

**Fig. 2:**
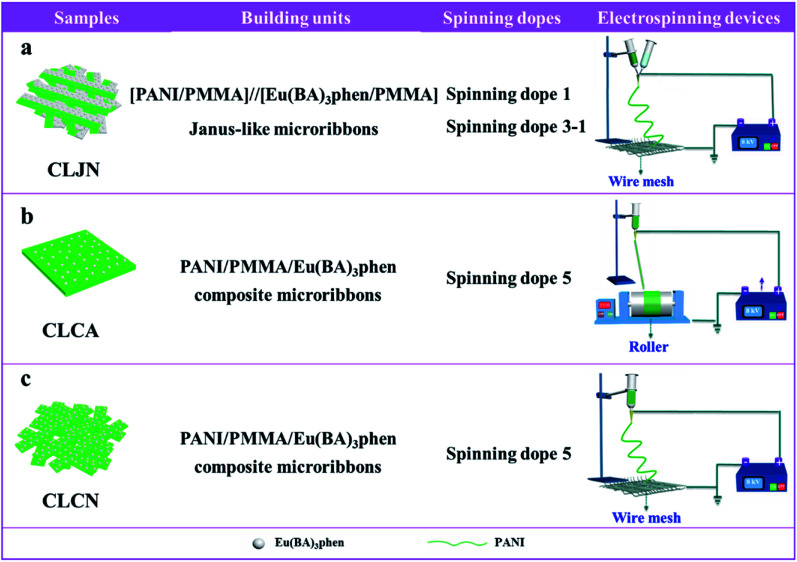
Schematic diagram of the fabrication of CLJN (a), CLCA (b) and CLCN (c).

**Fig. 3:**
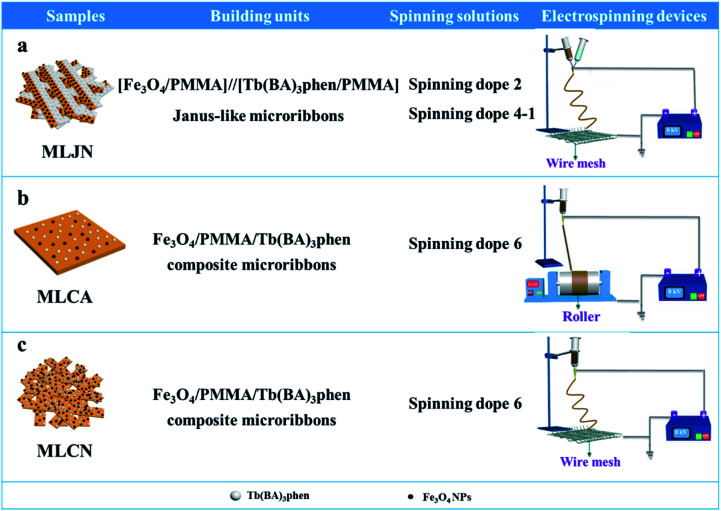
Schematic diagram of the fabrication of MLJN (a), MLCA (b) and MLCN (c).

Three comparative samples were prepared to reveal the benefits of CL layer in CML Janus-like pellicle. First, the wire mesh was used as a collector to gain [PANI/PMMA]//[Eu(BA)_3_phen/PMMA] Janus-like microribbons non-array (marked as CLJN) with other experimental conditions and parameters maintained as those for preparing CL layer. PANI/PMMA/Eu(BA)_3_phen composite microribbons array (named as CLCA) and PANI/PMMA/Eu(BA)_3_phen composite microribbons non-array (denoted as CLCN) were obtained by respectively selecting a roller and a piece of wire mesh as collectors when the spinning dope 5 was applied, and other experimental conditions and related parameters were the same as those for preparing the CL layer. The electrospinning devices for preparing the three comparative samples were illustrated in Fig. 2. The thicknesses of CLJN, CLCA and CLCN respectively were 186 μm, 180 μm and 184 μm. Similarly, other three comparative samples were prepared in order to explain the superiority of ML layer in CML Janus-like pellicle. Firstly, the wire mesh was used as a collector to obtain [Fe_3_O_4_/PMMA]//[Tb(BA)_3_phen/PMMA] Janus-like microribbons non-array (marked as MLJN) and other experimental parameters were maintained as those for preparing ML layer. Fe_3_O_4_/PMMA/Tb(BA)_3_phen/PMMA composite microribbons array (named as MLCA) and Fe_3_O_4_/PMMA/Tb(BA)_3_phen/PMMA composite microribbons non-array (denoted as MLCN) were obtained by respectively selecting the roller and a piece of wire mesh as collectors when spinning dope 6 was utilized, and other experimental parameters were the same as those for fabricating ML layer. The electrospinning devices for preparing the three comparative samples were illustrated in Fig. 3. The thicknesses of MLJN, MLCA and MLCN respectively were 197 μm, 183 μm and 189 μm.

### Characterization techniques

The characterization methods were described in the ESI.†

## Results and discussion

### Morphology and structure

**Fig. 4:**
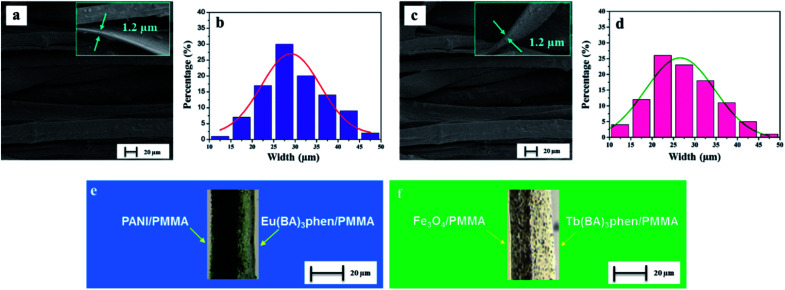
SEM images of CL layer (a) and ML layer (c) of CML Janus-like pellicle; width distribution histograms of Janus-like microribbon in CL layer (b) and in ML layer (d) of CML Janus-like pellicle; OM images of single [PANI/PMMA]//[Eu(BA)_3_phen/PMMA] Janus-like microribbon in CL layer (e) and single [Fe_3_O_4_/PMMA]//[Tb(BA)_3_phen/PMMA] Janus-like microribbon in ML layer (d) of CML Janus-like pellicle.

The morphology of CML Janus-like pellicle can be observed from SEM images. Fig. 4a shows the SEM image of CL layer of CML Janus-like pellicle. [PANI/PMMA]//[Eu(BA)_3_phen/PMMA] Janus-like microribbons in CL layer have smooth surfaces and array along a common direction. In order to investigate the diameter distribution of all samples, Nano Measurer 1.2 software is used to measure diameters of 100 microribbons from SEM images when the mass percentage of PANI to PMMA is fixed as 15% and the mass ratio of Fe_3_O_4_ NPs to PMMA is designated as 1 : 1, and the results are analyzed with statistics. The width and thickness of [PANI/PMMA]//[Eu(BA)_3_phen/PMMA] Janus-like microribbons respectively are *ca.* 28.99 ± 0.84 μm (Fig. 4b) and 1.2 μm. Fig. 4c shows the SEM image of ML layer of CML Janus-like pellicle. [Fe_3_O_4_/PMMA]//[Tb(BA)_3_phen/PMMA] Janus-like microribbons in M-L layer are closely arranged along the same direction. The width and thickness of [Fe_3_O_4_/PMMA]//[Tb(BA)_3_phen/PMMA] Janus-like microribbons respectively are *ca.* 26.56 ± 0.63 μm (Fig. 4d) and 1.2 μm. The OM observation is demonstrated to testify the Janus-like structure of [PANI/PMMA]//[Eu(BA)_3_phen/PMMA] Janus-like microribbons in CL layer and [Fe_3_O_4_/PMMA]//[Tb(BA)_3_phen/PMMA] Janus-like microribbons in M-L layer. A clear interface is observed between approximately transparent Eu(BA)_3_phen/PMMA and the dark green PANI/PMMA, as shown in Fig. 4e. It proves that [PANI/PMMA]//[Eu(BA)_3_phen/PMMA] Janus-like microribbon has favorable Janus-like structure. As seen from Fig. 4f, a clear interface is observed between approximately transparent Tb(BA)_3_phen/PMMA and brown Fe_3_O_4_/PMMA, which indicates that [Fe_3_O_4_/PMMA]//[Tb(BA)_3_phen/PMMA] Janus-like microribbon is successfully prepared. Furthermore, it can be seen from the Fig. 4f that the Fe_3_O_4_ NPs are evenly distributed on one side of single [Fe_3_O_4_/PMMA]//[Tb(BA)_3_phen/PMMA] Janus-like microribbon in ML layer without obvious agglomeration, proving that the Fe_3_O_4_ NPs are well distributed. The related SEM images and OM images of contrast samples are given in the ESI.†

**Fig. 5:**
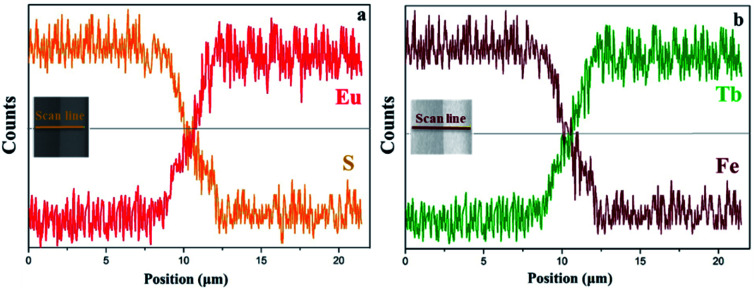
EDS line-scan analyses of [PANI/PMMA]//[Eu(BA)_3_phen/PMMA] Janus-like microribbon in CL layer (a) and [Fe_3_O_4_/PMMA]//[Tb(BA)_3_phen/PMMA] Janus-like microribbon in ML layer (b) of CML Janus-like pellicle.

**Fig. 6:**
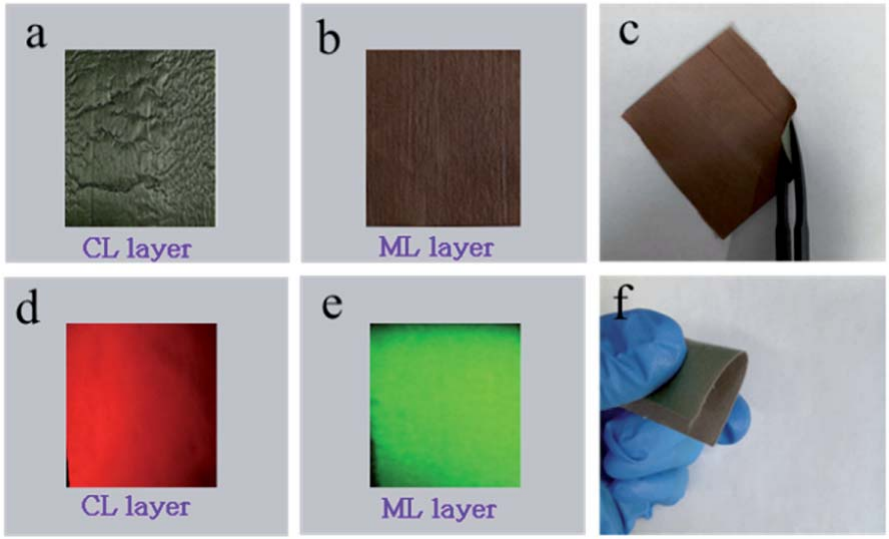
Physical digital photos of CL layer (a), ML layer (b) at unbent, CML Janus-like pellicle at bent (c and f) and digital camera photos of emission colors (under 290 nm light excitation in darkness) of CL layer (d) and ML layer (e) at unbent.

EDS line-scan analysis is carried out to futher demonstrate the Janus-like structure of [PANI/PMMA]//[Eu(BA)_3_phen/PMMA] Janus-like microribbon in CL layer and [Fe_3_O_4_/PMMA]//[Tb(BA)_3_phen/PMMA] Janus-like microribbon in ML layer. The results of EDS line-scan analysis for [PANI/PMMA]//[Eu(BA)_3_phen/PMMA] Janus-like microribbon are illustrated in Fig. 5a. The Eu and S elements symbolize severally Eu(BA)_3_phen and PANI. Eu is distributed on one side of single [PANI/PMMA]//[Eu(BA)_3_phen/PMMA] Janus-like microribbon and S arises on the other side, indicating that Eu(BA)_3_phen and PANI are respectively confined to specific regions in a Janus-like microribbon. The relevant results of [Fe_3_O_4_/PMMA]//[Tb(BA)_3_phen/PMMA] Janus-like microribbon are shown in Fig. 5b. The Tb and Fe elements symbolize respectively Tb(BA)_3_phen and Fe_3_O_4_ NPs. Tb is distributed in one side of single [Fe_3_O_4_/PMMA]//[Tb(BA)_3_phen/PMMA] Janus-like microribbon and Fe is dispersed in the other side, meaning that [Fe_3_O_4_/PMMA]//[Tb(BA)_3_phen/PMMA] Janus-like microribbon is successfully prepared. Physical digital photos of CML Janus-like pellicle are shown in Fig. 6a and b. The color of CL layer is blackish green and ML layer is brown. The surface of two layers of CML Janus-like pellicle is smooth and dense, besides, the Janus-like nanoribbons are parallelly and closely arranged in the same direction. Under 290 nm light excitation in darkness, CL layer and ML layer can respectively emit red luminescence and green luminescence (Fig. 6d and e). Fig. 6c and f prove CML Janus-like pellicle is flexible.

### Electrical conduction

**Fig. 7:**
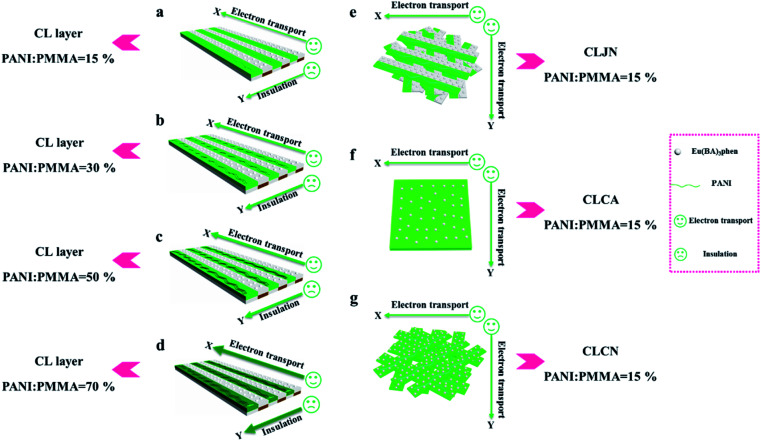
Schematic illustrations of conductive mechanism of CL layer (a–d), CLJN (e), CLCA (f) and CLCN (g).

**Fig. 8:**
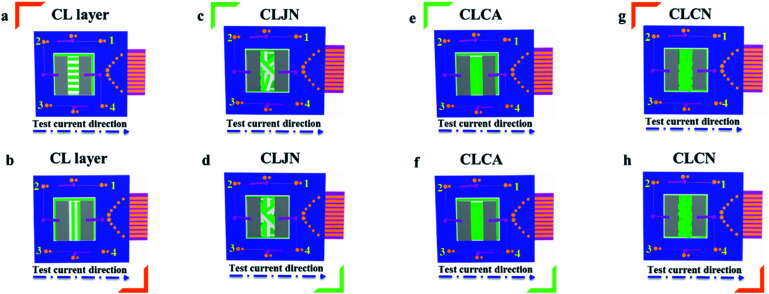
Schematic illustrations for conductance test of CL layer (a and b), CLJN (c and d), CLCA (e and f) and CLCN (g and h).

In order to easily explain the electrical conduction of all specimens, the *X* direction and *Y* direction (two perpendicular directions) are provided, as shown in Fig. 7. The direction along the nanoribbon is specified to *X* direction, while the direction perpendicular to nanoribbon is named as *Y* direction. CL layer containing conductive PANI can form a continuous conductive network along *X* direction, which can provide channels for electron transport. However, the continuous transmission of electrons is blocked along *Y* direction owing to the presence of the insulative materials, so CML Janus-like pellicle gains conductive aeolotropism resulting from the fact that the *X* direction of CL layer is conductive and the *Y* direction is insulative.^37^ The device schematics of conductance test are displayed in Fig. 8. Two pieces of tin sheets (1 × 0.45 cm^2^ for each one) are acted as the two electrodes, and separately pasted on the surface of CL layer and three comparative contrast samples with the area of 1 × 1 cm^2^ along *X* direction (Fig. 8a, c, e and g) and *Y* direction (Fig. 8b, d, f and h) by using conductive resin. The distance between the two electrodes is 0.1 cm. Table 3 summarizes the conductance of CL layer doped with variable amount of PANI and three contrast samples. *C*_*X*_ and *C*_*Y*_ respectively represent the conductance of *X* direction and *Y* direction and the ratio of *C*_*X*_ to *C*_*Y*_ (*C*_*X*_/*C*_*Y*_) is used to indicate conductive aeolotropism. For CML Janus-like pellicle, CL layer can form continuous conductive network along *X* direction to provide channels for electron transport, leading to the fact that the electron transport capacity is enhanced and the conductance is increased as increase of PANI. *C*_*X*_ changes from 2.17 × 10^−7^ S to 2.81 × 10^−2^ S and *C*_*Y*_ is almost unchanged, while the conductive aeolotropism (*C*_*X*_/*C*_*Y*_) changes from 1.18 × 10^3^ to 1.33 × 10^8^. CML Janus-like pellicle can obtain adjustable conductive aeolotropism by changing the doping amount of PANI and the highest conductive aeolotropism ratio can reach *ca.* 10^8^ when PANI content is 70%. The Janus-like microribbons in CL layer array along identical direction and the flow direction of electric current is the same along the *X* direction. Compared with CL layer, CLJN has weaker conductive aeolotropism, owing to the disordered arrangement of the Janus-like microribbons in CLJN and the disordered flow direction for electric current. Conductance decreases in both *X* direction and *Y* direction and further, *C*_*X*_ and *C*_*Y*_ are almost equal (*C*_*X*_/*C*_*Y*_ = 0.91, *ca.* 1). Compared with CL layer and CLJN, CLCA and CLCN have significantly lower conductance. This is because the Janus-like microribbons in CL layer and CLJN can realize micro-partition by effectively separating conductive material from insulative material, and PANI can form uninterrupted conductive networks, which can obtain the continuous transfer of electric current in the Janus-like microribbons. Nevertheless, the building units of CLCA and CLCN are composite microribbons where insulative materials are uniformly dispersed, which results in the fact that the continuous transfer of electric current is blocked and the conductance obviously reduces. CLCA has weak conductive aeolotropism (*C*_*X*_/*C*_*Y*_ = 10.9), which results from the fact that the interfaces among the microribbons in CLCA can become insulative media.^37^ However, the insulative ability of these interfaces is weak. For CLCN, the conductive PANI and insulative substances are mixed together in the composite microribbons with disorderly arrangements, so the conductance of CLCN is the lowest and CLCN does not have conductive aeolotropism (*C*_*X*_/*C*_*Y*_ = 0.95, *ca.* 1). The above results demonstrate that Janus-like microribbons can effectively separate conductive materials from insulating materials and limit them to specific regions, which can effectively reduce negative interferences between the two types of substances.

**Table 3:** Conductance of CL layer doped with different percentages of PANI and three contrast samples

Samples	PANI : PMMA (mass percentage)	Conductance (S)			Aeolotropic degree
		*C*_*X*_	*C*_*Y*_	*C*_*X*_/*C*_*Y*_	
CL layer	15%	2.17 × 10^−7^	1.84 × 10^−10^	1.18 × 10^3^	Medium
CL layer	30%	4.62 × 10^−5^	2.15 × 10^−10^	2.15 × 10^5^	Strong
CL layer	50%	1.64 × 10^−3^	1.37 × 10^−10^	1.20 × 10^7^	Very strong
CL layer	70%	2.81 × 10^−2^	2.12 × 10^−10^	1.33 × 10^8^	Very strong
CLJN	15%	1.16 × 10^−7^	1.27 × 10^−7^	0.91	None
CLCA	15%	2.21 × 10^−9^	2.01 × 10^−10^	10.9	Weak
CLCN	15%	1.97 × 10^−10^	2.07 × 10^−10^	0.95	None

**Fig. 9:**
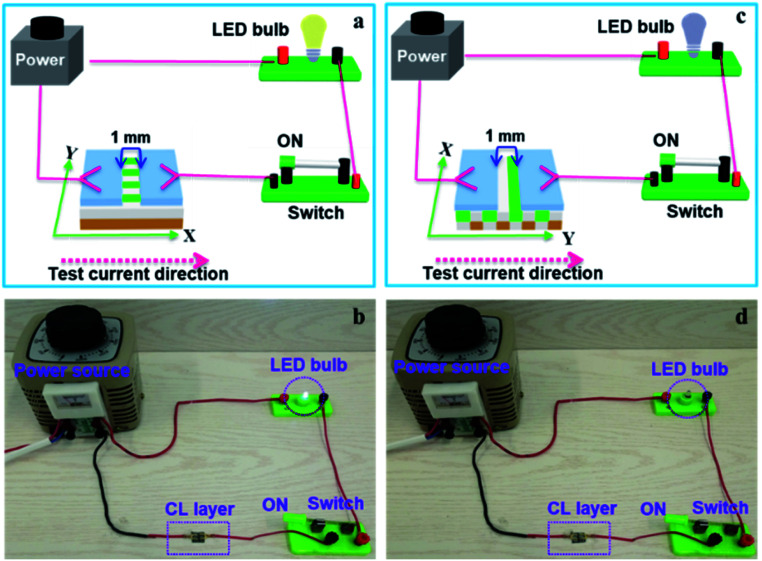
Schematic diagrams (a and c) and physical maps (b and d) for electrical demonstration of CL layer with the test current direction along *X* direction (a and b) and *Y* direction (c and d).

The above specimens are also used to carry out the electrical demonstration to further study the conductivity of CL layer in CML Janus-like pellicle. The schematic diagrams and physical maps of experimental devices are shown in the Fig. 9. A LED bulb (12 watt) is used as an indicator light, and a transformer is used as a power source. The voltage value is fixed as 25 V and cannot exceed 30 V during the demonstration. When the test current is transmitted paralleling to the length direction (*X* direction) of [PANI/PMMA]//[Eu(BA)_3_phen/PMMA] Janus-like microribbons in CL layer, the electron transportation is successive due to the existence of continuous PANI conductive network. The LED bulb lightens when the switch is closed, as illustrated in Fig. 9a and b. When the transmitted direction of test current is perpendicular to [PANI/PMMA]//[Eu(BA)_3_phen/PMMA] Janus-like microribbons (*Y* direction), the insulative materials including rare earth compounds and PMMA can block the electron transport so the LED bulb cannot be lighted up when the switch is turned on, as illustrated in Fig. 9c and d. The video of electrical demonstration (see the Video†) further proves this phenomenon. The electrical demonstration is performed for all CL layers when the mass percentage of PANI to PMMA changes from 15%, 30%, 50% to 70%. When the mass percentage of PANI to PMMA is 50% and 70% in CL layer, the LED bulb can be lighted up due to the high content of PANI. However, the LED bulb cannot be lighted up when the mass percentage of PANI to PMMA is 15% and 30% due to low PANI content and the weak conductivity of CL layer. Above results show that CML Janus-like pellicle has excellent conductivity and conductive aeolotropism.

### Crystallography and magnetism

**Fig. 10:**
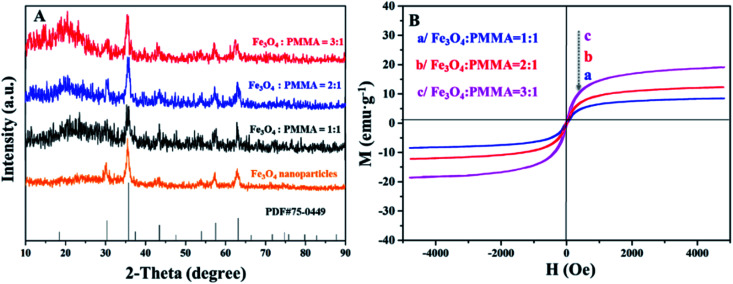
XRD patterns of Fe_3_O_4_ NPs and ML layer doped with different ratios of Fe_3_O_4_ NPs (A) and hysteresis loops of ML layer doped with different ratios of Fe_3_O_4_ NPs (B).

Fig. 10A demonstrates the XRD patterns of Fe_3_O_4_ NPs and ML layer doped with different amounts of Fe_3_O_4_ NPs. The diffraction peaks of Fe_3_O_4_ NPs can well match the standard card (PDF # 75-0449) of Fe_3_O_4_ with the cubic phase, and there is no apparent diffraction peaks of impurities, proving that Fe_3_O_4_ NPs with pure-phase structure are successfully synthesized. Meanwhile, different Fe_3_O_4_ NPs contents are successfully introduced into ML layer. The hysteresis loops and saturation magnetizations of ML layer doped with different ratios of Fe_3_O_4_ NPs are obtained and respectively provided in Fig. 10B and Table 4. The saturation magnetizations of ML layer gradually increase with the augment of doping quality of Fe_3_O_4_ NPs for the CML Janus-like pellicle, as seen in Table 4. It indicates that the magnetism of the CML Janus-like pellicle is adjustable by changing the doping amount of Fe_3_O_4_ NPs. The related XRD patterns of contrast samples are given in the ESI.†

**Table 4:** Saturation magnetizations of ML layer doped with different ratios of Fe_3_O_4_ NPs

Samples	Saturation magnetization (*M*_s_)/(emu g^−1^)
ML layer (Fe_3_O_4_ : PMMA = 3 : 1)	19.04
ML layer (Fe_3_O_4_ : PMMA = 2 : 1)	12.29
ML layer (Fe_3_O_4_ : PMMA = 1 : 1)	6.21

**Fig. 11:**
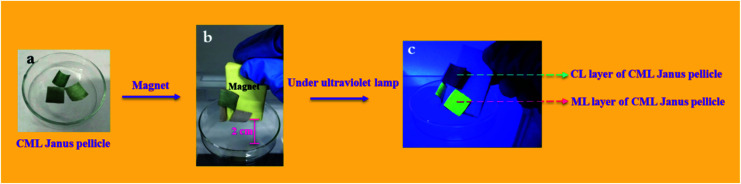
Physical maps for magnetic demonstration of CML Janus-like pellicle doped with different ratios without a magnet (a), with a magnet (b) and with a magnet and an ultraviolet lamp irradiation (c).

To further explore the magnetism of CML Janus-like pellicle, a magnetic demonstration is performed, and the physical maps of experimental devices are shown in the Fig. 11. CML Janus-like pellicle doped with different ratios of Fe_3_O_4_ NPs is cut into an area of 1 × 1 cm^2^ and put into a glass evaporator, as shown in Fig. 11a. Next, a magnet is placed above CML Janus-like pellicle and distances the bottom of glass evaporator away 2 cm. It is found that CML Janus-like pellicles can be attracted by the magnet (Fig. 11b), at the same time, CL layer can emit red luminescence while ML layer can emit green luminescence when irradiated by an ultraviolet lamp, as shown in Fig. 11c. This result not only further proves the strong magnetism of CML Janus-like pellicles, but also demonstrates the luminescence-magnetism difunctionality of CML Janus-like pellicles.

### Luminescence

**Fig. 12:**
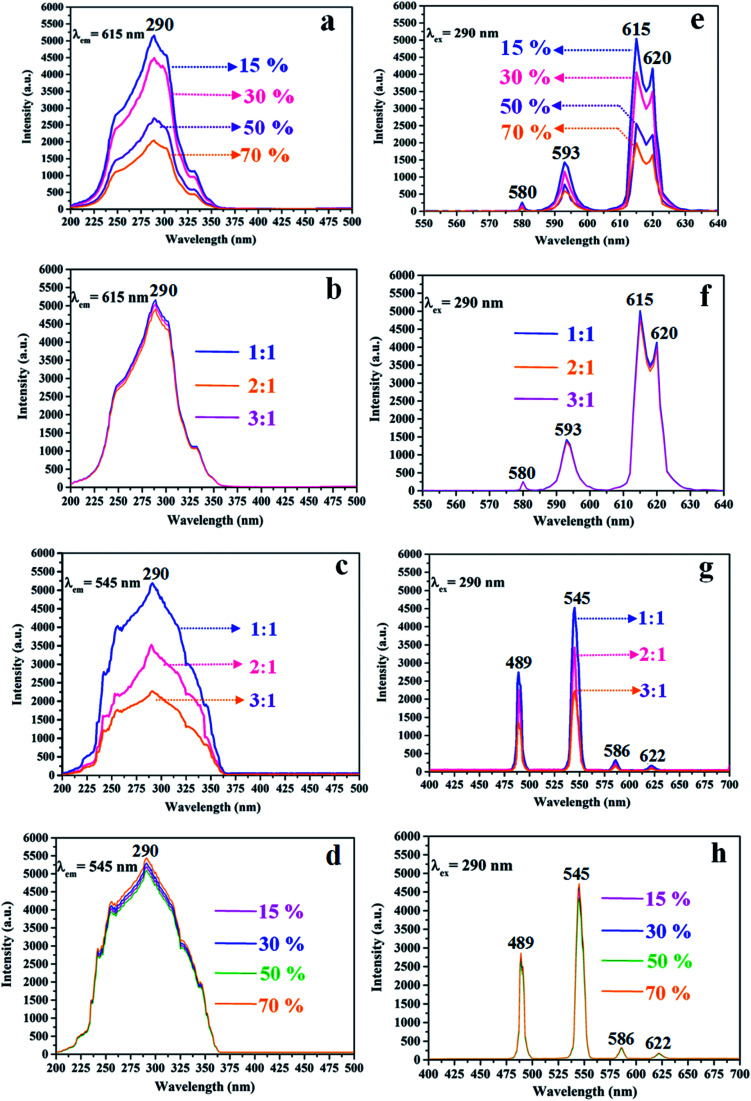
Excitation (a–d) and emission (e–h) spectra of CL layer doped with different percentages of PANI (a and e), CL layer with different ratios of Fe_3_O_4_ NPs to PMMA in ML layer when the mass percentage of PANI to PMMA is 15% (b and f), the ML layer doped with different ratios of Fe_3_O_4_ NPs (c and g) and ML layer with different percentages of PANI in CL layer when the mass ratio of Fe_3_O_4_ NPs to PMMA is 1 : 1 (d and h).

**Fig. 13:**
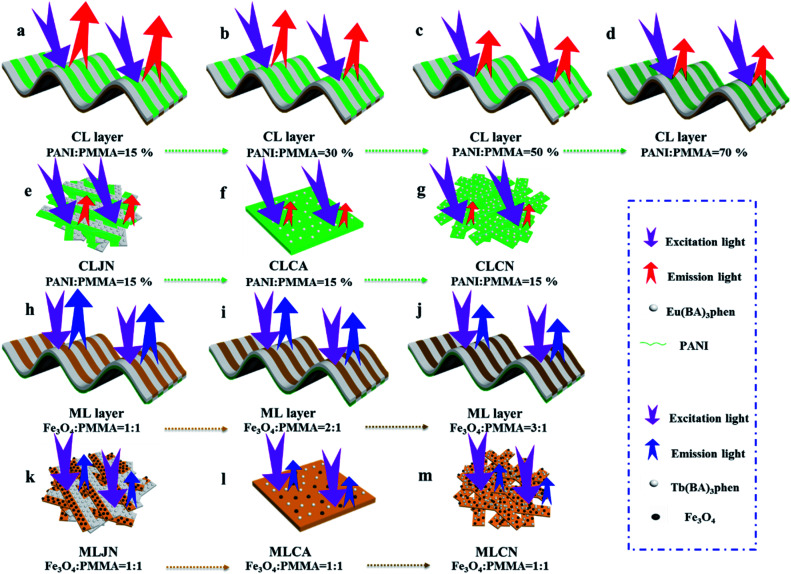
Schematic illustrations of excited and emitted lights in CL layer doped with different percentages of PANI (a–d), CLJN (e), CLCA (f), CLCN (g), ML layer doped with different ratios of Fe_3_O_4_ NPs (h–j), MLJN (k), MLCA (l) and MLCN (m).

**Fig. 14:**
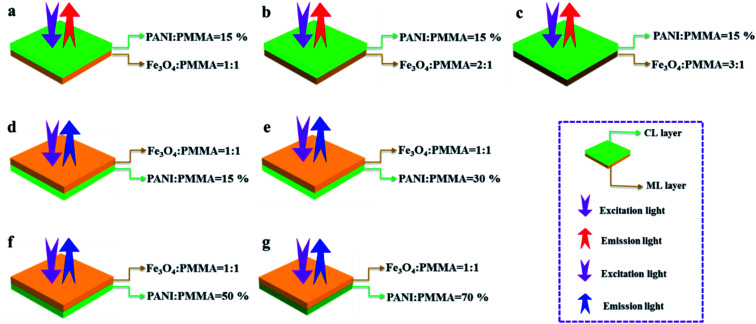
Schematic illustrations of excited and emitted lights of CL layer with different ratios of Fe_3_O_4_ NPs in ML layer when the mass percentage of PANI to PMMA is 15% (a–c) and ML layer with different percentages of PANI in CL layer when the mass ratio of Fe_3_O_4_ NPs to PMMA is 1 : 1 (d–g).

The variety of luminescence intensity of CL layer in CML Janus-like pellicle is explored when the mass percentage of PANI to PMMA changes from 15%, 30%, 50% to 70%. As found out from Fig. 12a and e, the strongest peak at 290 nm assigned to π → π* electron transition of ligands can be seen when the monitoring wavelength is 615 nm, and characteristic emission peaks seated at 580, 593, 615, 620 nm are gained when the excitation wavelength is 290 nm. They are originated from ^5^D_0_ → ^7^F_0_ (580 nm), ^5^D_0_ → ^7^F_1_ (593 nm), ^5^D_0_ → ^7^F_2_ (615 nm and 620 nm) energy level transitions of Eu^3+^, and ^5^D_0_ → ^7^F_2_ hypersensitive transition results in the strongest red light (615 nm). The luminescence intensity of CL layer gradually decreases with the increase of PANI. This is because PANI can absorb a portion of excited and emitted lights, and the absorption becomes more obvious as the more PANI introduced.^41^ The schematic illustrations of excited and emitted lights in CL layer with changeable percentages of PANI is also proved in order to explain this change, as revealed in Fig. 13a–d. Meanwhile, the color of CL layer gradually deepens with the augment of PANI content. The characteristic emission peaks of Tb^3+^ are not monitored in emission spectrum of CL layer. To further explore the influence of the ML layer on the luminescence of the CL layer, the luminescence of CL layer is measured when the mass ratio of Fe_3_O_4_ NPs to PMMA changes from 1 : 1, 2 : 1 to 3 : 1 in ML layer. As shown in Fig. 12b and f, there is no significant change in the luminescence of CL layer with variant ratios of Fe_3_O_4_ NPs in ML layer when the mass percentage of PANI to PMMA is fixed as 15%, indicating that the change of magnetic Fe_3_O_4_ NPs in ML layer has no obvious effect on the luminescence of CL layer. The schematic illustrations of excited and emitted lights in CL layer with different ratios of Fe_3_O_4_ NPs in ML layer further well illustrate this situation, as exhibited in Fig. 14a–c.

Similarly, the mass ratio of Fe_3_O_4_ NPs to PMMA changes from 1 : 1, 2 : 1 to 3 : 1 to explore the change of luminescence intensity of ML layer in CML Janus-like pellicle. As exhibited in Fig. 12c and g, the strongest peak (290 nm) is originated to π → π* electron transition of ligands when the monitoring wavelength is 545 nm, and characteristic emission peaks situated at 489, 545, 586, 622 nm coming from ^5^D_4_ → ^7^F_6_ (489 nm), ^5^D_4_ → ^7^F_5_ (545 nm), ^5^D_4_ → ^7^F_4_ (586 nm) and ^5^D_4_ → ^7^F_3_ (622 nm) energy level transitions of Tb^3+^ are obtained when the excitation wavelength is 290 nm. ^5^D_4_ → ^7^F_5_ hypersensitive transition leads to the strongest green light (545 nm). The luminescence intensity of ML layer decreases with the increase of Fe_3_O_4_ NPs and the color gradually deepens, as illustrated in Fig. 13h–j. These results are attributed to the fact that brown Fe_3_O_4_ NPs can absorb part of excited and emitted lights and the absorption is more obvious with the increase of Fe_3_O_4_ NPs,^40,51^ leading to the reduction of luminescence intensity. The characteristic emission peaks of Eu^3+^ are not found from emission spectrum of ML layer. Further, in order to research the impact of the CL layer on the luminescence of the ML layer, the luminescence of ML layer is determined when the mass percentage of PANI to PMMA changes from 15%, 30%, 50% to 70% in CL layer. As seen from Fig. 12d and h, no significant change in the luminescence of ML layer with different percentages of PANI in CL layer can be observed when the mass ratio of Fe_3_O_4_ NPs to PMMA is 1 : 1. It reveals that the change of conductive PANI in CL layer has no obvious influence on the luminescence of ML layer. The schematic illustrations of excited and emitted lights in ML layer with changeable percentages of PANI in CL layer further explicate this phenomenon, as illustrated in Fig. 14d–g. From the above-results, it is found that no apparent interferences between the two layers are found, fully demonstrating that the macroscopic partition achieved by using Janus-like structure of pellicle can efficaciously avoid negative interferences among different properties.

**Fig. 15:**
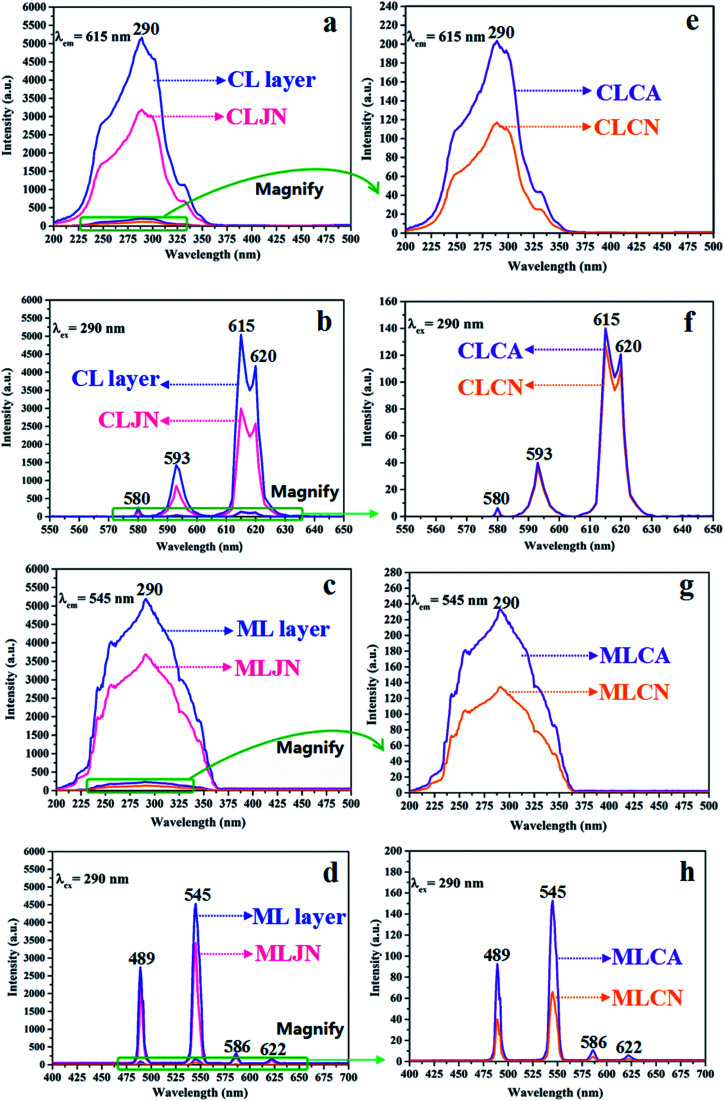
Excitation (a–d) and emission (e–h) spectra of CL layer and CLJN (a and b), CLCA and CLCN (e and f), ML layer and MLJN (c and d), MLCA and MLCN (g and h).

The luminescence intensities of CLJN, CLCA and CLCN are compared with that of CL layer of CML Janus-like pellicle, and the excitation and emission spectra are described in Fig. 15a, e, b and f. Compared to CL layer, CLJN has weaker luminescence intensity. The Janus-like microribbons on the surface of CL layer are arranged in a highly neat order, and the surface of CL layer is dense. Almost no excited light can get into the lower microribbons through the uppermost microribbons when the excited light arrives at the surface of the pellicle, and the emitted light is hardly absorbed. However, the surface of CLJN is porous owing to the loose and disorderly arrangement of Janus-like microribbons. Some excited light can transmit into the lower microribbons through the uppermost microribbons. In this case, the uppermost luminescent materials of the pellicle cannot be completely excited and the emitted light originated from the lower microribbons is also absorbed by the upper microribbons, so CLJN has weaker luminescence intensity compared to CL layer. CLCA has stronger luminescent intensity than CLCN, the reason is the same as that for CL layer and CLJN. The luminescence intensities of CLCA and CLCN obviously reduce contrasted to CL layer. For CL layer, the building unit is Janus-like microribbon that can effectively separate dark substance (PANI) from luminescent substance and effectively reduce the absorption of excited and emitted lights from PANI. For CLCA and CLCN, the building units are composite microribbons, where dark PANI and luminescent substance are evenly distributed in the same area, leading to the fact that more excited and emitted lights can be absorbed by PANI. By comparison, composite microribbons in CLCN are disorderly, so CLCN has the lowest luminescence intensity. The distinct schematic illustrations of excited and emitted lights in different contrast samples are detailed in Fig. 13e, f and g, and further explain the phenomenon. The luminescence intensities of MLJN, MLCA and MLCN are compared with that of ML layer of CML Janus-like pellicle, as described in Fig. 15c, g, d and h. Compared to ML layer, MLJN has weaker luminescence intensity, which is due to the loose and disorderly arrangement of Janus-like microribbons. MLCA has weaker luminescence intensity compared to ML layer and MLJN. The dark-colored Fe_3_O_4_ NPs and Tb(BA)_3_phen are evenly distributed in the composite microribbons, which results in the consequences that the absorption of excited and emitted lights is exacerbated and luminescence intensity of MLCA is reduced evidently. By comparison, MLCN has the lowest luminescence intensity owing to disorderly arrangement of composite microribbons. The distinct schematic illustrations of excited and emitted lights in different contrast samples are detailed in Fig. 13k–m, and further explain the phenomenon. The above results confirm that Janus-like microribbons, realizing microscopic partition, can effectively separate luminescent matters from dark matters and limit them to specific regions, which can greatly reduce the deleterious interferences between the two types of substances. From what has been discussed above, we can surely arrive at the conclusion that both microscopic partition and macroscopic partition can effectively reduce negative interferences among different properties. The design strategy of combining microscopic partition with macroscopic partition can not only ensure the independence of each excellent property, but also achieve a high degree of integration of all properties with little mutual interferences, and thus, is a practical and novel route to construct multifunctional materials.

## Conclusions

In summary, a new Janus-like pellicle, functionalized by conductive aeolotropism, magnetism and luminescence (defined as CML Janus-like pellicle) with top-bottom structure, is constructed by combining microcosmic partition with macroscopic partition *via* electrospinning. [PANI/PMMA]//[Eu(BA)_3_phen/PMMA] Janus microribbons and [Fe_3_O_4_/PMMA]//[Tb(BA)_3_phen/PMMA] Janus microribbons are respectively selected as building units to construct electrical conduction-luminescence layer (named as CL layer) and luminescence-magnetism layer (marked as ML layer). CL layer is found to have stronger red light emission compared to contrast samples. Furthermore, CL layer has tuned and strong aeolotropic conduction, and the electrical demonstration shows that C-L layer has good conductivity and the prepared materials have potential applications in flexible nanodevices. ML layer synchronously has strong green light emission and adjustable magnetism. Magnetic demonstration further proves the prepared CML Janus-like pellicle has strong magnetism and luminescence-magnetism difunctionality. Janus-like structure of the pellicle can obtain macroscopic partition and Janus-like microribbons can achieve microcosmic partition by separating luminescent substances from dark-colored Fe_3_O_4_ NPs and PANI. The different layers (CL layer and ML layer) of CML Janus-like pellicle have almost no interferences on electrical conduction, magnetism and luminescence. Such multifunctional materials can be used in flexible nanodevices, electromagnetic shielding, biological imaging and other fields.

## Conflicts of interest

There are no conflicts of interest to declare.

## Supplementary Material

RA-009-C9RA06444C-s001

RA-009-C9RA06444C-s002
