# Supplementary material for: Electrospun Janus-like pellicle displays coinstantaneous tri-function of aeolotropic conduction, magnetism and luminescence
Source: RSC Adv. 2019 Sep 30;9(53):30890–904. doi: 10.1039/c9ra06444c (PMC9072224; doi:10.1039/c9ra06444c)
Supplement: RA-009-C9RA06444C-s001 [file RA-009-C9RA06444C-s001.pdf]

Electronic Supplementary Material (ESI) for RSC Advances.
This journal is © The Royal Society of Chemistry 2019

# Supporting Information

# Electrospun Janus-like Pellicle Displays Coinstantaneous Tri-function of Aeolotropic Conduction, Magnetism and Luminescence

Yunrui Xie, Qianli Ma, Haina Qi, Yan Song, Jiao Tian, Makiyyu Abdullahi Musa, Wensheng Yu, Xiangting Dong*, Dan Li, Guixia Liu

Key Laboratory of Applied Chemistry and Nanotechnology at Universities of Jilin Province, Changchun University of Science and Technology, Changchun 130022

*Corresponding author: Fax: 86 0431 85383815; Tel: 86 0431 85582574; E-mail: dongxiangting888@163.com

## Experimental Sections

### Chemicals:

$Eu_2O_3$ (99.99 %), $Tb_4O_7$ (99.99 %), concentrated nitric acid, benzoic acid (BA), 1,10-phenanthroline (phen), anhydrous ethanol, benzoylperoxide (BPO), aniline (ANI), (1S)-(+)-camphor-10 sulfonic acid (CSA), ammonium persulfate (APS), $FeSO_4 \cdot 7H_2O$, $FeCl_3 \cdot 6H_2O$, $NH_4NO_3$, polyethylene glycol (PEG, Mr≈20 000), ammonia, oleic acid (OA), methylmethacrylate (MMA), *N,N*-dimethylformamide (DMF), $CHCl_3$. Deionized water was homemade. All the chemicals were analytically pure reagents and directly used as received without further purification.

### Characterization

The morphologies and structures of the samples were tested *via* a field emission scanning electron microscope (FESEM, XL-30) equipped with an energy-dispersive X-ray spectroscopy

(EDS). An optical microscope (OM, CVM500E) was employed to obtain the images of the samples. The conductive determinations were conducted on a Hall Effect measurement system (ECOPIA HMS-3000). The luminescence of the specimens was analyzed by using a Hitachi fluorescent spectrophotometer F-7000. The crystallographic information was gained by X-ray diffractometer (XRD, Bruker D8 FOCUS) by using a Cu Kα radiation source. The magnetism of the samples was detected by a vibrating sample magnetometer (VSE, MPMS SQUID XL). All tests were carried out at room temperature.

**Morphology and structure**

**Fig. S1a** is the SEM image of CLJN, where [PANI/PMMA]//[Eu(BA)$_3$phen/PMMA] Janus-like microribbons are disordered and the surface of microribbons is smooth. The width and thickness of [PANI/PMMA]//[Eu(BA)$_3$phen/PMMA] Janus-like microribbons in CLJN respectively are *ca.* 21.43±0.72 μm **(Fig. S2a)** and 1.3 μm. **Fig. S1b** and **Fig. S1c** respectively are SEM images of CLCA and CLCN. PANI/PMMA/Eu(BA)$_3$phen composite microribbons are aligned in the same direction and the width and thickness respectively are *ca.* 19.33±0.41 μm (**Fig. S2b**) and 1.3 μm. In CLCN, PANI/PMMA/Eu(BA)$_3$phen composite microribbons are disordered, and the width and thickness of respectively are *ca.* 19.07±0.41 μm (**Fig. S2c**) and 1.3 μm. In MLJN (**Fig. S1d**), [$Fe_3O_4$/PMMA]//[Tb(BA)$_3$phen/PMMA] Janus-like microribbons are disordered and the surface of microribbons is slightly rough. The width and thickness of [$Fe_3O_4$/PMMA]//[Tb(BA)$_3$phen/PMMA] Janus-like microribbons in MLJN respectively are *ca.* 19.30±0.85 μm (**Fig. S2d**) and 1.3 μm. **Fig. S1e** and **Fig. S1f** respectively are SEM images of MLCA and MLCN. It can been seen that almost $Fe_3O_4$/PMMA/Tb(BA)$_3$phen/PMMA composite microribbons in MLCA are aligned in the same direction and the width and thickness respectively are *ca.* 20.54±0.23 μm (**Fig. S2e**) and 1.3 μm. In MLCN, the surface of disordered $Fe_3O_4$/PMMA/Tb(BA)$_3$phen/PMMA composite

microribbons is slightly rough, and the width and thickness of respectively are *ca.* 18.32±0.25 μm (**Fig. S2f**) and 1.3 μm.

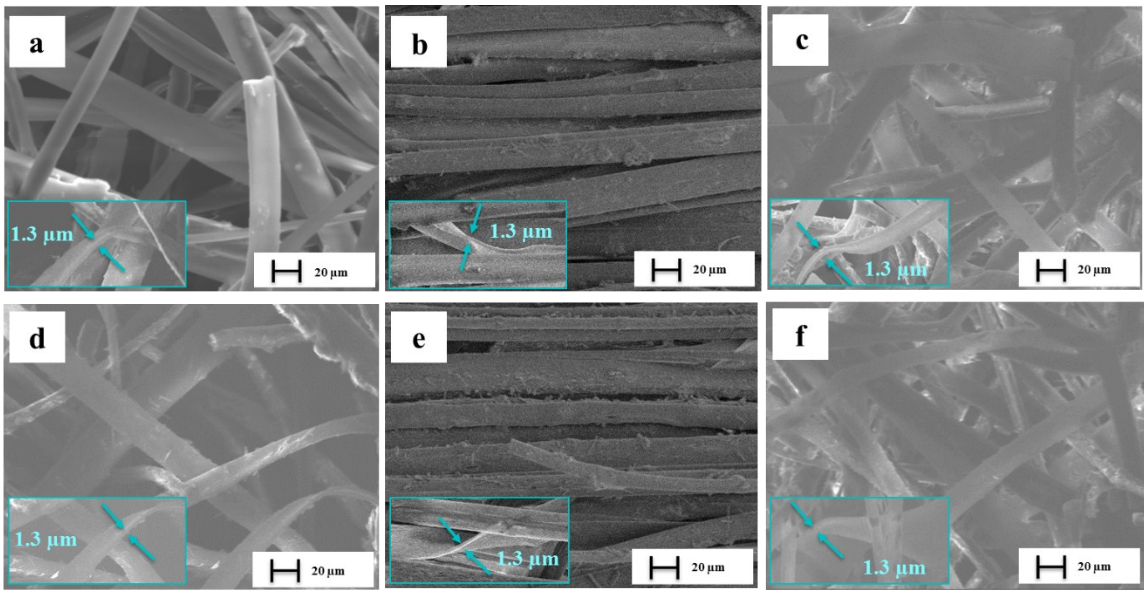


**Fig. S1** SEM images of CLJN (a), CLCA (b), CLCN (c), MLJN (d), MLCA (e) and MLCN (f)

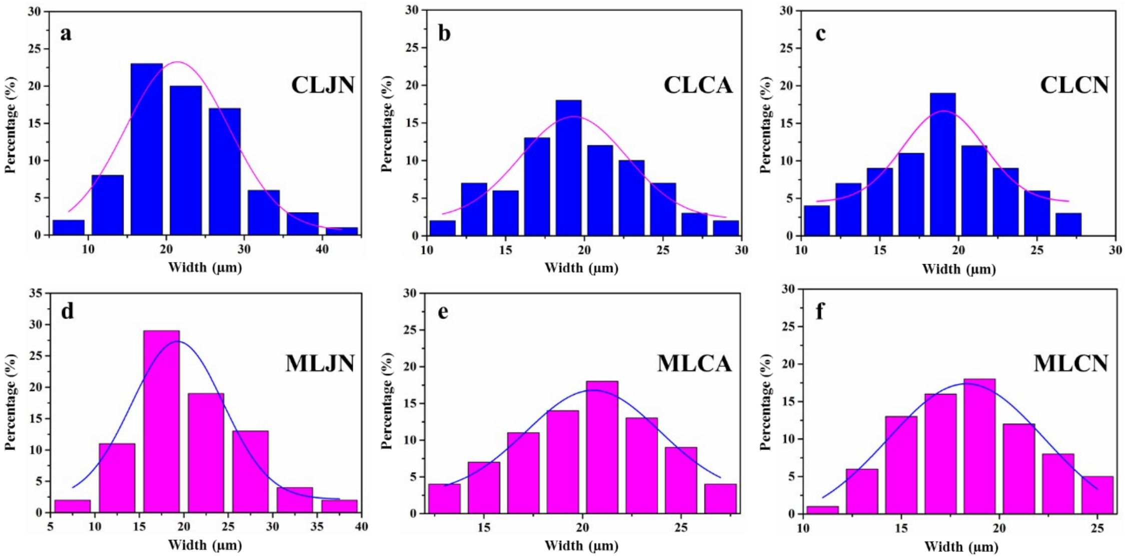


**Fig. S2** Width distribution histograms of CLJN (a), CLCA (b), CLCN (c), MLJN (d), MLCA (e) and MLCN (f)

A clear interface is observed between approximately transparent $Eu(BA)_3phen$/PMMA and the dark green PANI/PMMA, as shown in **Fig. S3a**. It proves that [PANI/PMMA]//[$Eu(BA)_3phen$/PMMA] Janus-like microribbon has favorable Janus-like structure in CLJN. However, no a clear interface in PANI/PMMA/$Eu(BA)_3phen$ composite microribbons for CLCA (**Fig. S3b**) and CLCN (**Fig. S3c)** is observed. As seen from **Fig. S3d**, a clear interface is observed between approximately transparent $Tb(BA)_3phen$/PMMA and brown $Fe_3O_4$/PMMA, which indicates that [$Fe_3O_4$/PMMA]//[$Tb(BA)_3phen$/PMMA] Janus-like microribbon is successfully prepared in MLJN. For MLCA (**Fig. S3e**) and MLCN (**Fig. S3f)**, no apparent interface is found in $Fe_3O_4$/PMMA/$Tb(BA)_3phen$/PMMA composite microribbons. These results show that Janus-like microribbon can achieve micro-zoning and reduce interferences among different functional substances. However, composite microribbon cannot achieve micro-zoning and cause harmful influences among different properties.

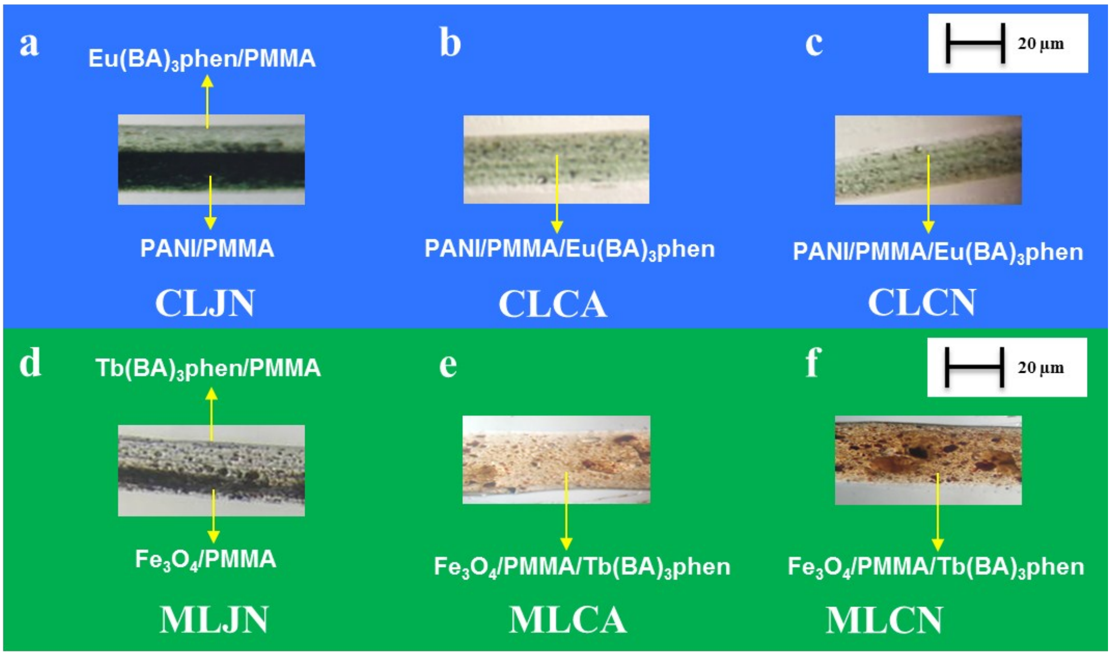


**Fig. S3** OM images of single [PANI/PMMA]//[$Eu(BA)_3phen$/PMMA] Janus-like microribbon in CLJN (a), single PANI/PMMA/$Eu(BA)_3phen$ composite microribbon in CLCA (b), single PANI/PMMA/$Eu(BA)_3phen$ composite microribbon in CLCN (c), single

[$Fe_3O_4$/PMMA]//[Tb(BA)$_3$phen/PMMA] Janus-like microribbon in MLJN (d), single $Fe_3O_4$/PMMA/Tb(BA)$_3$phen/PMMA composite microribbons in MLCA (e) and single $Fe_3O_4$/PMMA/Tb(BA)$_3$phen/PMMA composite microribbon in MLCN (f)

**Crystallography**

**Fig. S4** demonstrates the XRD patterns of ML layer, MLJN, MLCA and MLCN doped with the same amounts of $Fe_3O_4$ NPs ($Fe_3O_4$:PMMA=1:1). The diffraction peaks of MLJN, MLCA and MLCN can well match the standard card (PDF # 75-0449) of cubic phase of $Fe_3O_4$ and ML layer. No diffraction peaks of impurities are detected, indicating that pure-phase $Fe_3O_4$ NPs are successfully introduced into ML layer and three contrast samples.

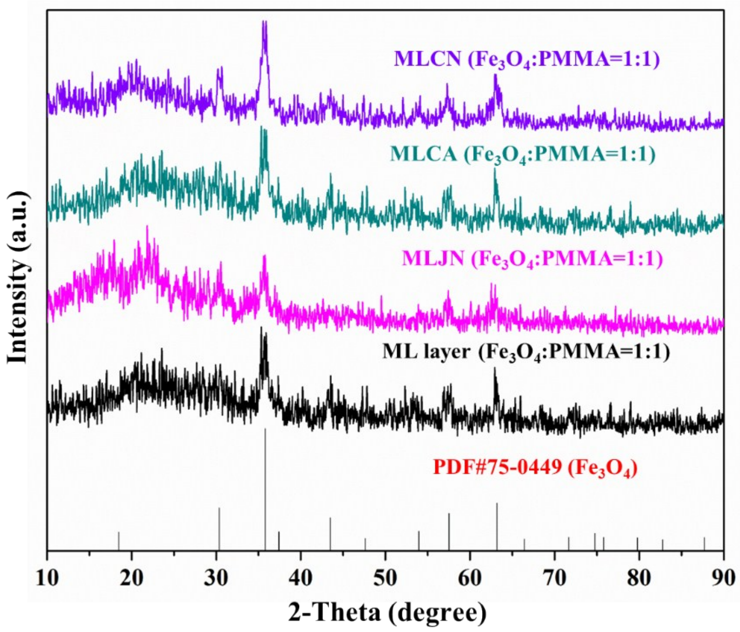


**Fig. S4** XRD patterns of ML layer, MLJN, MLCA and MLCN doped with the same amounts of $Fe_3O_4$ NPs ($Fe_3O_4$:PMMA=1:1)
